# Phenotype-Agnostic Molecular Subtyping of Neurodegenerative Disorders: The Cincinnati Cohort Biomarker Program (CCBP)

**DOI:** 10.3389/fnagi.2020.553635

**Published:** 2020-10-08

**Authors:** Andrea Sturchio, Luca Marsili, Joaquin A. Vizcarra, Alok K. Dwivedi, Marcelo A. Kauffman, Andrew P. Duker, Peixin Lu, Michael W. Pauciulo, Benjamin D. Wissel, Emily J. Hill, Benjamin Stecher, Elizabeth G. Keeling, Achala S. Vagal, Lily Wang, David B. Haslam, Matthew J. Robson, Caroline M. Tanner, Daniel W. Hagey, Samir El Andaloussi, Kariem Ezzat, Ronan M. T. Fleming, Long J. Lu, Max A. Little, Alberto J. Espay

**Affiliations:** ^1^James J. and Joan A. Gardner Family Center for Parkinson’s disease and Movement Disorders, Department of Neurology, University of Cincinnati, Cincinnati, OH, United States; ^2^Division of Biostatistics and Epidemiology, Department of Biomedical Sciences, Paul L. Foster School of Medicine, Texas Tech University Health Sciences Center, El Paso, TX, United States; ^3^Consultorio y Laboratorio de Neurogenética, Centro Universitario de Neurología “José María Ramos Mejía” y División Neurología, Hospital JM Ramos Mejía, Facultad de Medicina, Universidad de Buenos Aires, Buenos Aires, Argentina; ^4^Programa de Medicina de Precision y Genomica Clinica, Instituto de Investigaciones en Medicina Traslacional, Facultad de Ciencias Biomédicas, Universidad Austral– Consejo Nacional de Investigaciones Científicas y Técnicas de Argentina, Pilar, Argentina; ^5^Division of Biomedical Informatics, Cincinnati Children’s Hospital Medical Center, Department of Pediatrics, University of Cincinnati, Cincinnati, OH, United States; ^6^School of Information Management, Wuhan University, Wuhan, China; ^7^Division of Human Genetics, Cincinnati Children’s Hospital Medical Center, Department of Pediatrics, University of Cincinnati, Cincinnati, OH, United States; ^8^Department of Radiology, University of Cincinnati Medical Center, Cincinnati, OH, United States; ^9^Division of Infectious Diseases, Center for Inflammation and Tolerance, Cincinnati Children’s Hospital Medical Center, Cincinnati, OH, United States; ^10^Division of Pharmaceutical Sciences, James L. Winkle College of Pharmacy, University of Cincinnati, Cincinnati, Cincinnati, OH, United States; ^11^Department of Neurology, Weill Institute for Neurosciences, Parkinson’s Disease Research Education and Clinical Center, San Francisco Veteran’s Affairs Medical Center, University of California, San Francisco, San Francisco, CA, United States; ^12^Department of Laboratory Medicine, Clinical Research Center, Karolinska Institutet, Stockholm, Sweden; ^13^Analytical Biosciences, Division of Systems Biomedicine and Pharmacology, Leiden Academic Centre for Drug Research, Leiden University, Leiden, Netherlands; ^14^School of Computer Science, University of Birmingham, Birmingham, United Kingdom; ^15^Media Lab, Massachusetts Institute of Technology, Cambridge, MA, United States

**Keywords:** biomarkers, Parkinson’s disease, Alzheimer’s disease, neurodegeneration, cohort, drug repurposing, bioassay

## Abstract

Ongoing biomarker development programs have been designed to identify serologic or imaging signatures of clinico-pathologic entities, assuming distinct biological boundaries between them. Identified putative biomarkers have exhibited large variability and inconsistency between cohorts, and remain inadequate for selecting suitable recipients for potential disease-modifying interventions. We launched the Cincinnati Cohort Biomarker Program (CCBP) as a population-based, phenotype-agnostic longitudinal study. While patients affected by a wide range of neurodegenerative disorders will be deeply phenotyped using clinical, imaging, and mobile health technologies, analyses will not be anchored on phenotypic clusters but on bioassays of to-be-repurposed medications as well as on genomics, transcriptomics, proteomics, metabolomics, epigenomics, microbiomics, and pharmacogenomics analyses blinded to phenotypic data. Unique features of this cohort study include (1) a reverse biology-to-phenotype direction of biomarker development in which clinical, imaging, and mobile health technologies are subordinate to biological signals of interest; (2) hypothesis free, causally- and data driven-based analyses; (3) inclusive recruitment of patients with neurodegenerative disorders beyond clinical criteria-meeting patients with Parkinson’s and Alzheimer’s diseases, and (4) a large number of longitudinally followed participants. The parallel development of serum bioassays will be aimed at linking biologically suitable subjects to already available drugs with repurposing potential in future proof-of-concept adaptive clinical trials. Although many challenges are anticipated, including the unclear pathogenic relevance of identifiable biological signals and the possibility that some signals of importance may not yet be measurable with current technologies, this cohort study abandons the anchoring role of clinico-pathologic criteria in favor of biomarker-driven disease subtyping to facilitate future biosubtype-specific disease-modifying therapeutic efforts.

## Introduction

We have long assumed that the neuropathological findings of aggregated α-synuclein (α-syn) into Lewy bodies and Lewy neurites define and cause Parkinson’s disease (PD) and that aggregations of amyloid (Aβ) into plaques and tau into neurofibrillary tangles define and cause Alzheimer’s disease (AD), and that the distribution of these proteins explains their clinical heterogeneity (Espay et al., 2020). These pathological findings are, however, ubiquitous and do not correlate with agnostic post-mortem analysis: α-syn, Aβ, and tau aggregation are frequent “co-pathologies” in AD and PD (Irwin et al., 2017; Boyle et al., 2018; Karanth et al., 2020) and can be found even in super-survivors without dementia or parkinsonism (Head et al., 2009; Wallace et al., 2019). The overlapping pathological features may instead reflect clinical characteristics shared by PD and AD (Scarmeas et al., 2004, 2005; Kehagia et al., 2010). Indirect evidence from human studies suggest protein aggregation in sporadic cases may in fact be protective and not capable of discriminating clinical disease subtypes (Espay et al., 2019). As a result, it has become imperative to transition from the century-old, clinico-pathological convergent model on which diseases are classified to a systems biology framework, in which genotype and biomolecular abnormalities, rather than clinical phenotypes alone, define nosology and drive therapeutics (Espay et al., 2017).

Given these premises, we have recently launched at the University of Cincinnati’s James J. and Joan A. Gardner Center for Parkinson’s Disease and Movement Disorders, a phenotype-agnostic biomarker-discovery program aimed at characterizing biological subtypes of neurodegenerative disorders, particularly those best suited for targeting with therapies available for repurposing. This cohort study has unique features compared to ongoing [e.g., Parkinson’s Progression Marker Initiative (PPMI)] or newly assembled cohorts [e.g., Luxembourg study, Personalized Parkinson Project (PPP)] (Table 1) (Kenneth et al., 2011; Hipp et al., 2018; Bloem et al., 2019). The main novelty for our cohort study is a design based on the assumption we do not know which biomarkers have clinical relevance at the individual level. Accordingly, the recruitment will be deliberately inclusive of different neurodegenerative phenotypes with the expectation that biological subtypes may not align with clinico-pathological subtypes.

**TABLE 1:** Comparison between established biomarker-development cohorts.

	**PPMI (Kenneth et al., 2011)**	**Luxembourg (Hipp et al., 2018)**	**PPP (Bloem et al., 2019)**	**CCBP**
Overview	To identify biomarkers to define progression of PD (independent variable: PD; dependent variable: biological measures)	To identify biomarkers to define progression of PD and APD (independent variable: PD, APDs; dependent variable: biological measures)	To identify biomarkers to define progression of PD (independent variable: PD; dependent variable: biological measures)	To identify biomarkers of subtypes regardless of clinical diagnosis (independent variable: biological signals; dependent variable: phenotypes)
Objectives	Use clinical assessments of short-term (6 months) progression to predict long-term PD progression Define longitudinal stability of PD subtypes (TD vs. PIGD vs. indeterminant)	Clinico-genomic stratification Progression of PD and APD APD differential diagnosis Characterize cognitive, gait, and vision disturbances	Compare treatment response with biomarker status Association between biomarkers and progression of motor and cognitive symptoms	Define causal biological disease pathways Identify subpopulations with biological outliers Repurpose existing therapies to target subpopulations
Inclusion criteria	PD ≤ 2 years Positive DAT-scan	PD, APD (any disease stage) ≥ 18 years old	PD ≤ 5 years ≥ 18 years old	PD and PD-like, AD and AD-like disorders (inclusive, any disease stage) ≥ 18 years old
Sample size	1400* patients, 200 healthy controls	800 patients, 800 healthy controls	650 patients, no healthy controls	4,000 patients, 1,000 healthy controls
Data analysis	Hypothesis-driven analysis: standard exploratory analyses	Data-driven: standard statistical and machine learning approaches for classification, regression, clusterization, and time-series analyses	Hypothesis-driven analysis: linear prediction models	Data-driven: iterative causal modeling and deep learning
Directionality of analysis	Phenotype-to-biomarker	Phenotype-to-biomarker	Phenotype-to-biomarker	Biomarker-to-phenotype
Follow-up period	Every 6 months (∼6 years accrued to date)	Annual	Up to 2 years	Annual (for at least 5 years)
In clinic- assessment	Motor symptoms Non-motor symptoms + neuropsychological assessment Demographics and epidemiological data	Motor symptoms Non-motor symptoms + neuropsychological assessment ADLs questionnaires Sensor-based: gait analysis Demographics and epidemiological data	Motor symptoms Non-motor symptoms + neuropsychological assessment ADLs questionnaires Sensor-based: continuous accelerometer recording, pulse rate, and ECG data Demographics and epidemiological data	Motor symptoms Non-motor symptoms + neuropsychological assessment ADLs questionnaires Sensor-based: motor and voice analysis Demographics and epidemiological data
Biospecimens	DNA, plasma, serum CSF, urine	DNA, RNA, peripheral blood mononuclear cells, plasma, serum; CSF, stools, fibroblast, and colon biopsy are optional	DNA, RNA, peripheral blood mononuclear cells, plasma, serum, CSF, stools	DNA, RNA, peripheral blood mononuclear cells, plasma, serum, urine, stools, exosomes
Neuro-imaging	DaT-scan and MRI imaging	Based on patient records (if available)	MRI (including fMRI)	MRI (including 3D, DTI, and fMRI)
At-home assessment	Smartwatch data recording: accelerometer data, pulse rate, ECG	Mobile phone application: finger tapping and gait analysis	2-years continuous smartwatch data recording: accelerometer data, pulse rate, ECG, clinical scales, and questionnaires	24-h continuous smartwatch data recording: pulse rate, HRV, sleep-related parameters

PPMI, Parkinson’s Progression Markers Initiative; PPP, Personalized Parkinson Project; CCBP, Cincinnati Cohort Biomarker Program; PD, Parkinson’s disease; APD, atypical parkinsonian disorders (PSP, CBS, MSA); AD, Alzheimer disease; TD, tremor dominant; PIGD, postural instability and gait disturbance; ADLs, activities of daily living; RNA, ribonucleic acid; DNA, deoxyribonucleic acid; CSF, cerebrospinal fluid; DaT-scan, Dopamine Transporter Scan; MRI, magnetic resonance imaging; fMRI, functional MRI; ECG, electrocardiogram; HRV, heart rate variability. *Encompassing different cohorts of patients. PPMI 2.0 will soon increase the number of patients and ex the use of mobile health technologies.

Here we summarize the methodological aspects of this cohort study, including phenotypic measures and analytic approach, and discuss anticipated challenges.

## The Cincinnati Cohort Biomarker Program

This is an omics-based, longitudinal, structural causal model, non-phenotype-driven population-based study. We will enroll a total of 4,000 patients with neurodegenerative diseases and 1,000 healthy age-matched controls with yearly follow-up for at least 5 years, extended to 10 and beyond contingent on additional funding. At each visit, patients will undergo a similar clinical, paraclinical, and biospecimen collection. Pragmatic approaches such as streamlining data gathering (prioritizing biospecimen collection) will be allowed if important to retain subjects and minimize dropouts. The exploratory nature of this study rendered it unsuitable for funding considerations by agencies giving continued preference for hypothesis-based studies based on the prevailing clinico-pathologic model of neurodegenerative diseases, which remains the gold standard for nosology, biomarker validation, and disease modification. As a result, this study was funded through philanthropy, with major support by the James J. and Joan A. Gardner Family Foundation. The main aim is to identify biological outliers defining molecular disease subtypes, with a focus on those suitable for targeting with already available therapies (repurposing) in future built-in adaptive clinical trials.

### Inclusion and Exclusion Criteria

Given the inclusive nature of the study, we are recruiting subjects older than 18 years of age exhibiting a range of parkinsonisms representing PD and PD-like disorders, such as progressive supranuclear palsy, multiple system atrophy, and corticobasal syndrome, as well as AD and AD-like disorders, such as frontotemporal dementias, normal pressure hydrocephalus, and vascular dementia. The enrollment of young subjects could help in the identification of early biomarkers in specific conditions (e.g., genetic). However, our enrollment will be initially focused on the elderly population seeking care at the University of Cincinnati Gardner Center, which receives referrals from a wide range of Cincinnati-area neurologists. The Center evaluates a representative population of neurodegenerative disorders seeking care in the Cincinnati area. We will also recruit age- and sex-matched healthy controls. Controls that during the study assessment manifest signs of neurological disease will be shifted as cases.

Although “Cases” and “Controls” are determined by virtue of the presence or absence of neurological symptoms, respectively, our inclusion criteria for neurodegenerative disorders are otherwise deliberately inclusive, based on the premise that we do not *a priori* know in which clinical phenotypes will the first targetable molecular subtypes be identified. As noted in Section “Data Analysis and Management,” none of proposed analysis will use the classification of participants into cases or controls, nor any phenotypic subtype created therein, as independent variables. Nevertheless, all participants will be referred by a neurologist to make sure they present specific signs of parkinsonism or dementia. In case of doubt, the Principal Investigator will decide if the subjects fit inclusion criteria. Only subjects with recognized causes or contributors for their motor or cognitive manifestations (e.g., vitamin B12 deficiency) and those requiring aggressive medical management will be excluded.

### Ethics, Collection, and Storage of Biological Samples

The study protocol was approved by the Institutional Review Board of the University of Cincinnati (protocol number 2020-0039). Informed consent is obtained from all subjects with the conduct of the study fully adhering to the principles of the Declaration of Helsinki. Biospecimens will be collected from subjects and healthy controls, including peripheral blood, urine, and stool.

Plasma is being isolated from blood collected in EDTA vacutainers and aliquoted for future use, including isolation of plasma proteins and extracellular vesicles (EVs). As all cells secrete EVs, they are abundant in all bodily fluids and have been shown to carry diverse species of nucleic acids, proteins, and lipids (van Niel et al., 2018). Plasma will be subjected to size exclusion chromatography (70 nm qEV original, Izon Science) to separate EVs from soluble proteins. The EVs present in each sample will be quantified using nanoparticle tracking analysis (NanoSight NS300, Malvern Panalytical), and their surface proteins characterized by a flow cytometry method optimized for vesicle analysis (Wiklander et al., 2018). Following isolation, we will extract RNA and sequence the mRNA present within these vesicles using methods developed for single-cell RNA-sequencing. In order to amplify the most informative signals in total EVs mRNA, we will utilize known neuron, astrocyte and oligodendrocyte cell surface markers using immunoprecipitation (Miltenyi Biotec).

A urine sample is being collected in a sterile kit during in-clinic visits. Stool samples are aliquoted into preservative containers (OMNIgene.Gut, DNA Genotek, Corp.) immediately after passage. Samples are transferred to −80° storage within 72 h. DNA is subsequently extracted from 0.25 gm stool using the PowerFecal Pro extraction kit (Qiagen, Inc.). DNA sequencing libraries will be constructed (Nextera XT, Illumina, Corp.) and pooled for sequencing on an Illumina sequencing machine (NextSeq500, Illumina, Corp.). Sequencing reads will be aligned to a microbial genome database using Kraken (Wood and Salzberg, 2014) to determine the assemblage of microorganisms present in each fecal sample (Quigley, 2017). Biospecimens are processed and aliquoted for downstream use consistent with the strategy of future use/sharing of the samples. All sample meta-data are tracked via the DT Biobank’s LIMS system to catalog the chain of custody and processing details. Stool samples are stored at −80°C in the Microbial Genomics and Metagenomics Laboratory at Cincinnati Children’s Hospital. Participants are also asked to participate in an optional brain donation program.

Genomics, transcriptomics, proteomics, metabolomics, epigenomics, and microbiomics will be processed from our biological samples. We will use validated methods for the analysis of the samples to ensure feasibility and reproducibility of the study in future independent cohorts. The specific methods will be selected at a later time; this will give us greater flexibility in the choice of assays as the analytic technologies become less expensive. Also, we may add other ‘–omics’ (e.g., lipidomics, etc.) in the future.

### Clinical, Paraclinical, and Neuroimaging Assessments

#### Clinical Scales and Questionnaires

Motor and non-motor symptoms are assessed through the Movement Disorders Society Unified Parkinson’s Disease Rating Scale (MDS-UPDRS part II and III) (Goetz et al., 2008), the Tinetti Gait and Balance scale (Tinetti et al., 1986), the Non-motor Symptoms Scale (NMSS) (Chaudhuri et al., 2006), the Parkinson’s Disease Quality of Life Questionnaire (PDQ-8) (Jenkinson et al., 1997), the Epworth Sleepiness Scale (ESS) (Johns and Hocking, 1997), the Activities of Daily Living (ADL), the Instrumental ADL (iADL) (Katz, 1983), the Beck Depression Inventory scale (BDI) (Beck and Beamesderfer, 1974), Beck Anxiety Inventory scale (BAI) (Beck et al., 1988), the REM Sleep Behavior Disorder Screening Questionnaire (RBDSQ) (Stiasny-Kolster et al., 2007), and the Montreal Cognitive Assessment (MoCA) (Nasreddine et al., 2005). An extensive epidemiological, demographical, pharmacological, and lifestyle questionnaire, as well as a Food Frequency Questionnaire (FFQ),^1^ are also collected.

#### Gait and Postural Stability Outcome Measures Obtained Using Mobile Health Technologies

Gait and postural stability are measured in the following conditions (Axivity, Ltd., Newcastle upon Tyne, United Kingdom): (1) Two-minute Walk: Subjects are asked to walk a straight path for 2 min. Parameters include: stride length, gait speed, stride width, and stride asymmetry; (2) Instrumented Time Up and Go (iTUG): Subjects are instructed to sit comfortably in an armless chair. At the “go” signal, they rise from the chair without using support, walk 3 m, turn 180° and walk back; (3) Postural Sway: Subjects are asked to stand with their hands at their sides and feet together spaced by a wooden wedge on a firm surface; (4) 360° Turn: Subjects are instructed to turn in a complete circle (360°), first to the left, and then to the right. Other measures include: (1) Tapping test: Subjects are asked to tap on the smartphone screen for 30 s; (2) Rest and postural tremor tests: Subjects hold their arm out straight for 30 s, and subsequently rest their arms in the lap while counting down from 100; and (3) Voice and speech tests: Subjects are asked to say “*aaaah*” at a comfortable pitch and loudness, and subsequently recite a short, phonetically-balanced passage, into an Android-based smartphone microphone.

A 3-Tesla brain MRI will be obtained within 6 months from the baseline. A comprehensive protocol including 3D T1 fast spoiled gradient echo (FSPGR), 3D T2-weighted, 3D T2-FLAIR, susceptibility weighted imaging (SWI), resting state functional MRI (fMRI), diffusion tensor imaging (DTI), and 3D arterial spin labelling (ASL) will be performed. 3D T1 FSPGR sequence provides volumetric analysis of regional atrophy. T2 and FLAIR sequences will be analyzed for chronic small vessel disease including white matter disease, lacunar infarcts, dilated perivascular spaces. SWI will provide information on iron deposition in the deep nuclei and microbleeds. Resting state fMRI will be analyzed for changes in functional connectivity. DTI tractography analysis will provide information on white matter integrity.

### At Home Sensor-Based Assessment

Participants are provided with smartwatches (Sony Corporation, Tokyo, Japan) for at-home 24-h continuous collection of sensor data such as accelerometry and wrist-based photoplethysmography, from which estimates of multiple behavioral parameters, including sleep behavior quality, heart rate variability and step count will be obtained.

### Data Storage and Process

All biological samples are stored for future analyses in a dedicated Biobank at Cincinnati Children’s Hospital Medical Center (CCHMC) and Discover Together Biobank using established protocols, for processing, storage, and future analysis. The database was designed to account for the longitudinal study design, linkage to multi-omics measurements and formats, and capacity to store big data. The stored data are labeled according to processed or unprocessed data, methods, and type of omics data. All the samples are coded using an identifier reflecting sites and subject number. All the samples are preprocessed for background correction, quality control and standard deviation of the intensity ratios. Prior to conducting analyses, normalization using LOWESS or quantiles, scaling with baseline correction, outlier removal, and missing imputation for less than 20% missing data using K-nearest neighbor imputation will be performed. BioMart for database and Bioconductor for data processing and analyses will be used along with specific software required for sequence, network, reads, mining, and pathways will be utilized according to their specific purposes. We plan to create an online platform where de-identified and analyzed data can be shared. To protect confidentiality and prevent bias, all imaging data will be deidentified and transmitted with unique study identification numbers to the imaging core lab, utilizing a HIPAA complaint secure platform. Imaging readings will be recorded on electronic case report forms and integrated seamlessly with the clinical data.

### Data Quality Management

A pre-analytical standard operating procedure (SOP) has been developed. The multiple steps included are aimed at minimizing biases at forming and analyzing substudy cohorts. The following SOP are highlighted: (a) subjects are selected only by neurologists; (b) controls are selected from the same population and time period than cases; (c) a substantially large sample size will permit estimating rare molecular subtypes; (d) pragmatic assessments to minimize dropouts and maximize adherence to protocol over a long observational period. Finally, our interdisciplinary team is meeting regularly to review the quality controls of data collection, SOP protocol adherence, data-gathering issues, and concerns related to ethics, data storage, data process, and management.

## Data Analysis and Management

### Aim

The main aim is to identify biologically unique biological subgroups with emphasis on those suitable for repurposing of already available therapies using proof-of-concept adaptive clinical trials.

### Sample Size and Statistical Power

The sample size of this study was computed using several simulations under various conditions. We utilized the Qiu and Joe (2009) formula (10 × *d* × *k*) (Qiu and Joe, 2009) where *d* is the number of variables included for clustering while *k* is the number of clusters and formula (70 × *d*) (Dolnicar et al., 2014). Using this formula to estimate the moderate, adjusted Rand index values produces a sample size of 3500 with 50 biological markers. This sample size is powered for detecting at least 10 subtypes with 40 biological markers using the Qiu and Joe formula. Furthermore, a total of 800 healthy controls are required to form a comparative group based on 1:2 case-control design for detecting small Cohen effect sizes (*D* = 0.2) between groups with more than 90% power and 5% level of significance. The sample size suggested in this study is more than sufficient to detect small (odds ratio 1.2 or standardized mean difference 0.20) to moderate (OR = 1.5 or SMD = 0.50) expected associations between individual subtypes and clinical outcomes depending on the types of outcomes and subtypes with more than 80% power and 5% level of significance and covariates accounting for 10 to 50% of variance in a given outcome using logistic regression analysis. This sample size also ensures adequate power for detecting small to moderate Cohen’s effect sizes (SMD 0.20–0.50) using two-sided unpaired *t*-tests. The sample size estimation was also found to be sufficient using data-driven sample size driven algorithm (DSD) (Billoir et al., 2016) and sample size in high-dimensionality data settings using the MV power algorithm (Guo et al., 2010). We note here that these formulas depend upon assumptions (such as Gaussianity) which may not hold for these data and for the kinds of clustering analysis we plan to use in this study and can only be considered reasonable to justify the sample size. Although a sample size of 3500 patients and 800 healthy controls was estimated as sufficient, we plan to enroll 4000 cases and 1000 controls in order to account for potential dropouts. The sample size will most likely need to increase to identify heretofore unanticipated molecular subtypes.

#### Exploratory Data Analysis

All potential biomarkers will be compared between cases and controls using a bootstrap test to screen for significant biomarkers from each omics platform and thereafter we will apply Bayesian exponential family principal components analysis (BE-PCA) (Shakir et al., 2008), a generalization of principal component analysis (PCA), which is a widely used method of statistical analysis and simplification of data sets, to reduce the dimensionality of the multi-omic data (Wold et al., 1987). We avoid the use of simple PCA because some of the variables we measure in this project are likely to be non-Gaussian.

### Data-Driven Causal Inference

**FIGURE 1:**
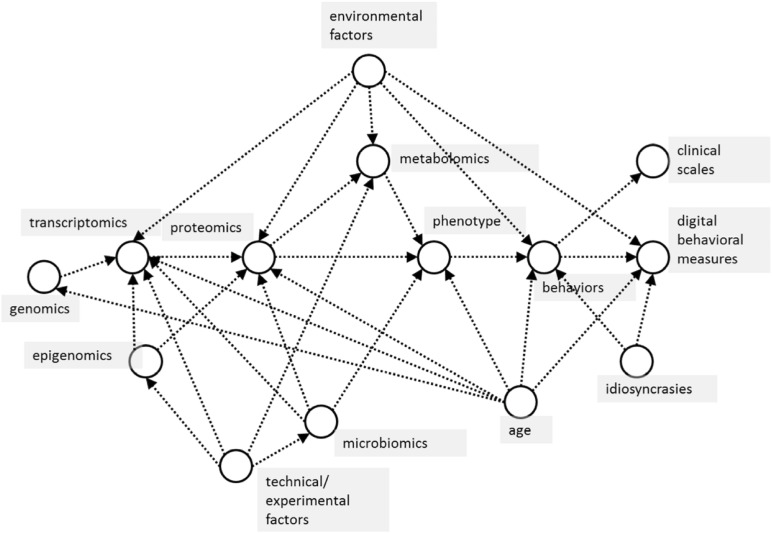
Basic causal model of proposed relationships between measured variables in the CCBP cohort study. Arrows between variables (in circles) indicate the dominant direction of causal influence between them. In this study, machine learning is used to model predictive relationships, but these should also be causal, not merely associational, relationships. For example, in predicting phenotype (effect) from omics data (causes), confounders such as subject age influence both cause and effect variables, which makes it critical to take these into consideration when using predictive machine learning algorithms.

The analytical approach of this study will be based on the latest techniques from statistics and data science (Little, 2019), revolving around *causal modeling and inference* of the interaction between all the variables captured in the study across genomics, transcriptomics, proteomics, metabolomics, epigenomics, microbiomics, and pharmacogenomics data (Figure 1). Starting with a simple causal model built using existing datasets, the model can be used for various purposes, including simulating randomized trials using causal inference, and acting as a guide to designing pragmatic trials to collect appropriate data to “fill in” missing information in the causal model. Results of these simulated trials will then further inform the modeling and statistical analysis choices, with the end goal of deriving a simple, mechanistic model that is both explanatory and predictive, which can be used to extract “subtypes” most likely to respond to therapies (Pearl, 2010).

The justification for the use of these techniques is that they aim to minimize the misleading effects of reliance on speculative and unproven theories of disease, behavior and symptom mechanisms while avoiding the problems of purely data-driven modeling, which can be easily confounded by unmeasured variables, poor-quality data or mischaracterized measurement processes.

These advances in causal inferential methods rely on a synthesis of two analytical techniques (Little and Badawy, 2019):

(1) *Data-driven approaches*. These approaches often have high predictive accuracy, and can capture high-dimensional, non-Gaussian, non-linear relationships. Machine learning is one example. The primary drawback is their limited explanatory power and high sensitivity to irrelevant confounding effects, which inevitably creep into measurements.
(2) *Causal modeling approaches.* A set of probabilistic relationships is drawn up to describe the mechanistic processes explaining the data. Because these models traditionally require fully-specified probabilistic relationships between variables, they often do not make quantitatively accurate predictions, but they do allow realistic, causal interactions among biological, behavioral and symptom expression processes to be built in to the analysis. This causal structure is essential in this study given the sheer number of variables and the resulting complexity of interaction between them.

We propose to use a synthesis of these two approaches, which can be described as data-driven causal inference. This aims to exploit the advantages of the high predictive accuracy of data-driven approaches and the realism of causal modeling. It respects the causal structure of the real world captured by the measurements, and is verified against the high-dimensional, non-Gaussian measured data with non-linear interactions, promising to circumvent both the problems of erroneous clinico-pathological reasoning and prevent data analysis which is heavily biased by spurious correlations because its structure can disentangle confounding factors in the measured data, for example.

Technically, data-driven causal inference involves finding variables and their covariates (Figure 1), isolating the mechanism predicting these variables using causal bootstrapping (Little and Badawy, 2019) or other causal adjustment methods (Pearl, 2010), then using the data to fit a predictive model of that isolated mechanism. The isolated mechanisms can then be assembled into a full, predictive causal network. After examining the associations of identified biological subtypes with clinical characteristics and outcomes, the severity of subtypes, their motor and non-motor functionalities, and progression pattern will be determined by integrating data from biological interpretation of subtypes as well. Visual interpretations obtained using Bayesian exponential family PCA and other dimensionality reduction techniques and relationship with clinical neurodegenerative disease subtypes, will be summarized to generate a global view of each subtype. The main benefit of this causal-inference data driven model is not the validation in separate populations but the identification of suitable candidates, within the cohort for future repurposing therapy approaches.

### Machine Learning-Based Subtyping and Integration

#### Subtyping Based on Individual Markers From Integrative Analysis

The analysis of the biological data should lead to clustering subjects with shared biomolecular alterations regardless of phenotype (Espay et al., 2017). In data-driven biological subtyping, the “truth” is unknown and the analysis hypothesis free. Clustering is a major method for disease subtyping based on high-dimensional omics data (Wang and Gu, 2016). We will apply clustering methods to identify subtypes in genomics, transcriptomics, proteomics, metabolomics, epigenomics, microbiomics, and pharmacogenomics. There are currently two main methods for the fusion clustering of multi-omics data [i.e., iCluster, similarity network fusion (SNF)] based on the sample similarity network. Studies have shown that SNF has better performance in disease subtyping than iCluster (e.g., cancer) (Wang et al., 2014; Wang and Gu, 2016). We will perform unsupervised clustering on the processed data by SNF and validate similarities and dissimilarities in identified subtypes using moCluster and pattern fusion analysis by adaptive alignment of multiple heterogeneous omics data. Because clustering analysis is an unsupervised learning method, the results cannot be tested by ground truth which usually indicates the accuracy of training set’s classification of supervised leaning techniques. We can also perform bioinformatics analysis, such as differential expression analysis and functional enrichment analysis, for different subtypes and compare the difference among them. Data-driven subtypes will be determined using various parameters described above. Deep phenotyping from clinical (e.g., development of clinical milestones such as falls, progression of motor and non-motor symptoms, etc.), paraclinical (e.g., mobile health technologies), and neuroimaging data (e.g., brain atrophy) will be used as outcome measures or dependent variables. The longitudinal design, with multiple follow-ups, will give us information about the casual role of potentially druggable biomarkers. The relationship between biomarkers and disease will require similar assessments in the control group.

#### Subtyping Based on Composite Markers From Integrative Analysis

The clustering of markers (joint expressions of important features) arising from different omics measurements may be useful in identifying unique subtypes of patients as opposed to using patterns of individual markers to form patient subtyping. This procedure typically involves a two-stage framework of clustering. The first stage of clustering groups the subset of variables into disjointed segments whereas the second stage creates subtyping of patients by exploring the patterns in the identified clusters of markers from the first stage. We will utilize unsupervised feature selection methods such as sparse partial least square (sPLS), sparse canonical correlation analysis (sCCA) (Witten and Tibshirani, 2009), and variable cluster analysis (VCLUS) in the first step followed by moCluster (Meng et al., 2016) and SNF in the second stage to determine subtypes.

#### Subtyping Based on Outlier and Non-Gaussian Markers

Heterogeneity may exit in the identified subtypes of patients. Generally, clustering approaches are conducted to determine subtypes and variable selection after removing outliers and non-Gaussian data. As opposed to removing outliers and non-Gaussian data, several unique subtypes and biological heterogeneity can be obtained by determining subtypes based on outlier markers. In this regard, two novel approaches can be adopted to identify outlier markers as well as non-normal markers. We will employ outlier profile and pathway analysis (OPPAR) using the modified cancer outlier profile analysis (mCOPA) (Wang et al., 2012). The mCOPA is used to identify markers that are outliers either up-regulated or down-regulated. We will also apply the maximum ordered subset *t*-statistics (MOST) (Karrila et al., 2011) method for identifying bimodal distributed markers. After selecting the appropriate set of non-normal and outlier markers, moCluster and SNF methods will be used to cluster patients into homogenous patterns of non-normal and outlier markers. These steps of identifying subtypes will be replicated for gene set enrichment analysis using OPPAR.

#### Subtyping Based on Dynamic Network Biomarkers

Individual sets of omics may have limitations, such as poor sample quality or data sparsity, network-based stratification can be used to overcome these limitations and identify unique patient subtypes. We will employ a network-based stratification approach for baseline omics data that determines patients with genes in similar network regions (Hofree et al., 2013). The dynamic network biomarkers (DNBs) method examines time-dependent alterations in biomarkers. We will select the cases-markers which are not statistically different at the baseline from controls and determine the longitudinal changes in the markers according to disease progression or treatment response. MoCluster and SNF will then be applied to determining subtypes based on the changes in non-significant markers.

### Bioassay Development for Currently Available Therapies

**FIGURE 2:**
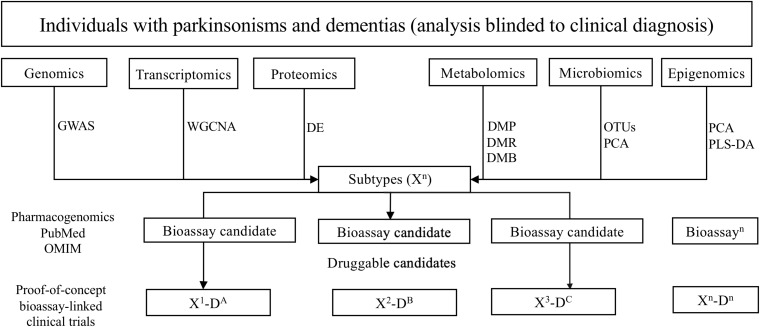
Overview of the pipeline to ascertaining biologically suitable subpopulations for drug repurposing. GWAS, genome-wide association study; WGCNA, weighted correlation network analysis; DE, differential expression analysis; DMP, differential methylation probe; DMR, differential methylation region; DMB, differential methylation block; PCA, principal component analysis; PLS-DA, partial least squares discrimination analysis; OUTs, operational taxonomic unit.

First, genomics, transcriptomics, proteomics, metabolomics, epigenomics, and microbiomics data will serve to identify potentially altered molecular pathways for each global neurodegenerative subtype (Figure 2). Bioassay candidates will be selected depending on candidates identified by relevant pathway analyses. For example, from the genomics data, we will perform genome-wide association study (GWAS) analysis to obtain SNPs of each subtype and then identify the potential pathogenic genotype and pathways in which they are associated. Viable bioassay candidates will be selected, determined by the generation of high-throughput clinically relevant assays for the quantification of expression and/or biologic state of candidates.

Second, online databases, including OMIM and PubMed, will be searched for related mechanistic information. Specifically, we will collect information of the effects of gain of function (GOF) and loss of function (LOF) in human and/or mammalian models and remove targets that can significantly aggravate the corresponding phenotype. Targets will be obtained through candidate analysis above and candidate drugs with repurposing potential will be recognized for future proof-of-concept clinical trials from identified pathways/protein combinations and drug-related protein information (Table 2).

**TABLE 2:** Available putative disease-modifying drugs for bioassay development (and targeted repurposing) examined in phase III clinical trials in Parkinson’s disease.

**Drug**	**Mechanism of action**	**Pathways for potential bioassay development**	
		**Increasing**	**Decreasing**
GLP 1 analogs (Baggio and Drucker, 2007; Li et al., 2012; Morales et al., 2014; Nassar et al., 2015; Athauda and Foltynie, 2016)	Anti-inflammatory, anti-apoptotic, mitochondrial enhancement	Bcl-2, Bcl-XL, Mfn2, SIRT1, PGC-1a, NGF, BDNF, ChAT activity	NF-Kb, iNOS, TNF- α, ICAM-1, MPO
Tocopherols (vitamin E) (Rizvi et al., 2014; Uchoa et al., 2016; Comitato et al., 2017)	Anti-oxidant, cells membrane protection, enhancement of immune system, regulation of cell proliferation	Tocopherols (α, β, γ, δ), tocotrienols (α, β, γ, δ), CD4, IL2, PI3K activation	GSSG/GSH, HMGCoA, HIF-1α, ICAM-1, VCAM-1, cAMP, MAPK/ERK, 12-lipoxygenase, arachidonic acid metabolism, oxidation of PUFA
Carnitine and Acetyl-L-carnitine (ALCAR) (Zanelli et al., 2005; Ferreira and McKenna, 2017)	Anti-oxidant, anti-inflammatory effects	pNFH, NGF	Free carnitine/acylated carnitine, GSSG/GSH, oxidation of PUFA, TNF- α
Pioglitazone (Swanson et al., 2011; Galimberti and Scarpini, 2017; Villapol, 2018; Xia et al., 2018)	Anti-oxidant, mitochondrial enhancement	COX-2, MMP9, SR-A, iNOS, Cu/Zn SOD, CD36, CD68, VEGF	STAT1, NF-κB, TNF-α, NADPH oxidase, LDH
Riluzole (Dennys et al., 2015)	Neurotropic	CT-1, BDNF, GDNF	
Selegiline – Rasagiline (Wu et al., 2015; Inaba-Hasegawa et al., 2017; Szökö et al., 2018)	Mitochondrial enhancement, anti-apoptotic, anti-inflammatory, anti-oxidant	Bcl-2, BDNF, NGF, GDNF	Nrf2, NF-κB, GSSG/GSH, oxidation of PUFA, oxidative pattern, NMDA-induced neurotoxicity
Minocycline (Thomas, 2004; Zemke and Majid, 2004)	Anti-oxidant, anti-apoptotic, anti-inflammatory, enhancement of immune system	IL10, PGE-2, Th-2	iNOS, NADPH-oxidase, caspase-1, caspase-3, IL1, COX-2, MMP2, MMP9, ICE, TNF- α, p38 MAPK phosphorylation, Th-1
Coenzyme Q10 (Fetoni et al., 2009; Orsucci et al., 2011; Fan et al., 2017)	Anti-inflammatory, anti-oxidant, anti-apoptotic	Complexes I + III and II + III activities, percentage of oxidized to total CoQ10, CoQ10/total cholesterol, mutations in ubiquinone biosynthetic genes (i.e., *PDSS1*, *PDSS2*, *CABC1*)	CRP, IL6, TNF- α, LDH, caspase 3
Mitoquinone (Analog CoQ10) (Ghosh et al., 2010; Orsucci et al., 2011; Zhou et al., 2018)	Anti-oxidant, mitochondrial enhancement	Heme oxygenase-1 NADPH, glutathione peroxidase, SOD	MDA, aconitase, oxidation of PUFA, ICAM-1
GPI 1485 (Harikishore and Sup Yoon, 2015)	Regulation of immune system, mediation of protein-protein interactions, mitochondrial enhancement, Neurotrophic	NGF, protective mitochondrial oxidative pathway	NF-AT, IL2, damage in mitochondrial DNA
AAV2-Neurturin (Olanow et al., 2015)	Neurotrophic	GDNF	N/A
GDNF (Liatermin) (Lang et al., 2006)	Neurotrophic	GDNF	N/A
Paliroden (Labie et al., 2006)	Neurotrophic	N/A	AChE, β-amyloid formation
PYM50028 (Cogane) (Visanji et al., 2008)	Neurotrophic	NGF, BDNF	N/A
Omigapil (TCH346 or CGP3466) (Olanow et al., 2006)	Anti-apoptotic	N/A	GAPDH
CEP-1347 (Parkinson Study Group PRECEPT Investigators, 2007; Kumar et al., 2015)	Anti-apoptotic	N/A	JNK signaling pathway
Deferiprone (Hider and Hoffbrand, 2018)	Iron chelation	N/A	Oxidation of dopamine and hydroxyl radicals, reduction NTBI
Isradipine (Ortner et al., 2017)	Antagonism of L-type Ca_v_1.3 calcium channels	N/A	Cyto-solic Ca^2+^ levels, mitochondrial oxidant stress

*NF-kB, nuclear factor kappa-light-chain-enhancer of activated B cells; iNOS, inducible nitric oxide synthase; TNF- α, tumor necrosis factor; ICAM-1, intercellular adhesion molecule; MPO, myeloperoxidase; PKA, protein kinase A; PI3K, phosphoinositide 3-kinase; Bcl-2, B-cell lymphoma 2; Bcl-XL, B-cell lymphoma-extra-large; Mfn2, mitofusin; SIRT1, Sirtuin 1; PGC-1a, peroxisome proliferator-activated receptor-gamma coactivator 1a; NGF, nerve growth factor; BDNF, brain-derived neurotrophic factor; GDNF, glial cell-derived neurotrophic factor; ChAT, choline acetyltransferase; HMGCoA, cholesterol biosynthesis by suppressing 3-hydroxy-3-methylglutaryl-CoA; VCAM-1, vascular cell adhesion protein 1; CD4, cluster of differentiation 4; IL2, interleukin2; cAMP, cyclic adenosine monophosphate; MAPK/ERK, Ras-Raf-MEK-ERK pathway; GSSG/GSH, oxidized glutathione, glutathione; HIF-1α, hypoxia-inducible factor 1-alpha; PUFA, polyunsaturated fatty acids; pNFH, phosphorylated high-molecular weight neurofilament; STAT1, signal transducer and activator of transcription 1; COX-2, cyclooxygenase-2; MMP2, metalloproteinase 2; MMP9, metalloproteinase 9; SR-A, scavenger receptor A; Cu/Zn SOD, copper/zinc superoxide dismutase; CD36, cluster of differentiation 36; CD68, cluster of differentiation 68; NADPH oxidase, nicotinamide adenine dinucleotide phosphate oxidase; VEGF, vascular endothelial growth factor; LDH, lactate dehydrogenase; CoQ10, coenzyme Q10; CT-1, cardiotrophin-1; Nrf2, nuclear factor E2-related factor 2; NMDA, *N*-methyl-D-aspartate; IL10, interleukin 10; IL1, interleukin 1; PGE-2, prostaglandin E2; ICE, interleukin converting enzyme; MMP, metalloproteinases; Th-1, T helper cells 1; Th-2, T helper cells 2; p38 MAPK, p38 mitogen-activated protein kinase; CRP, C-reactive protein; IL6, Interleukin 6; MDA malondialdehyde; NF-AT, nuclear factor of activated T-cells; AChE, acetylcholinesterase; GAPDH, glyceraldehyde-3-phosphate dehydrogenase; JNK, c-Jun N-terminal kinase; NTBI, non-transferrin bound iron; N/A, not available.*

We plan to work with industry partners to develop/utilize bioassays for the presumed mechanisms of actions for each of the candidate drugs. Given the phenotype-agnostic nature of this study, after the identification of bioassay-based abnormality suggesting vulnerability to a specific drug, a proof-of-concept clinical trial will be designed to match the drug with the bioassay-defined clinical cohort in order to evaluate for preliminary safety and efficacy of such to-be-repurposed intervention.

### Reliability and Validation of Patient Subtypes

Various approaches will be used to assess replicability, naturalness, and validation of cluster subtypes. The reliability will be assessed by the adjusted Rand statistic and percentage agreement on cross-validated hold-out testing. The validation will be assessed by comparing the clusters across different clustering methods (SNF, moCluster) (sPLA and sCCA) and (OPPAR followed by SNF, moCluster) and the concordance index (c-statistic) by evaluating the predictive performance of each cluster on primary outcomes across different methods.

## Anticipated Challenges

### Accessibility of Target Tissue and Use of Systemic Surrogates

Given the impossibility of serial brain biopsies, we can only rely on extra-cerebral surrogates (e.g., peripheral blood, urine, stool) to assess phenomena associated with brain neurodegeneration (Lehmann-Werman et al., 2016). Nevertheless, selected biological alterations associated with central nervous system neurodegeneration can also be detected in other tissues (Kaushik and Cuervo, 2015; Lehmann-Werman et al., 2016); for instance, EVs will be used as a platform for “liquid biopsies.” EVs have been shown to transport this molecular cargo directly between neighboring cells, as well as to distant cells via blood and other fluids (van Niel et al., 2018). EVs bear both surface proteins and intracellular contents from their parent cells into peripheral fluids, which are then accessible without the invasiveness of tissue biopsy (El Andaloussi et al., 2013). Moreover, in the future, EVs may also serve as a delivery system for therapies given that, as native nanoparticles, they benefit from immune tolerance and the ability to cross biological barriers (van Niel et al., 2018; Wiklander et al., 2019).

### Relevance of Biomarkers

Neurodegeneration starts years prior to symptom onset (Cacabelos, 2017). This creates difficulties in distinguishing between early biomarkers, related to causal disease mechanisms, and late biomarkers, possibly end results of other processes, themselves pathogenic, or resulting from response to various treatments (Espay et al., 2017). Moreover, early or late biomarkers may be transient or constant across neurodegenerative disorders, potentially underestimating or overestimating the importance of an early or late biomarker depending on the time of data acquisition. A population-based study design with control subjects, multiple visits, longitudinal assessments and next-generation statistical analysis may help mitigate these issues.

### Development of Bioassays

Some of the known mechanisms of therapies with repurposing potential (Table 2) may not be relevant to disease pathogenesis in any subtype, even if bioassays can be developed to measure their range in a laboratory. Some bioassay candidates can be difficult to deploy or measure with existing technology in a manner that would make them clinically viable. Connecting specific biomarkers to disease stage/progression will be difficult given our study design. This concern will be ameliorated by using promising bioassays to select patients for future proof-of-concept drug studies. Such studies will contribute toward separating primary from secondary biologic mechanisms of each neurodegenerative subtype.

### Uncertainty About Extent of Unknowns

While the data-driven design of this study favors the collection of data without *a priori* hypotheses for later analysis using discovery algorithms (Kim et al., 2016), a major challenge is to define which biologically promising targets may be more relevant than any of the currently known biomarkers. Also, some technologies may be insufficiently sensitive for potentially relevant biomarkers or result in false negative assays. As for the known variability of prior omics data, we expect that to be attenuated by the unbiased analysis, not anchored on diagnostic or phenotypic data. The creation of a robust biobank is designed to mitigate these difficulties by providing the opportunity to re-analyze samples and data in the future.

## The “All of Us” Program

The “All of US” program is an important effort funded by the NIH starting in 2015, aiming to collect clinical, paraclinical, and biological data in a very large population, not preselected for the presence of neurodegenerative disorders (All of Us Research Program Investigators, 2019). The goal of the program is to enroll at least 1 million persons nationwide from 340 recruitment sites (All of Us Research Program Investigators, 2019). This effort represents a significant step forward in the understanding of human health and disease. However, the lack of focus on neurodegenerative disorders (or any other disorder) represents an important limitation from the standpoint of our research objectives.

Compared to the “All of US,” our study aims to merge an “inclusive” approach to all neurodegenerative disorders and utilizes standardized clinical questionnaires and scales, in-clinic and at-home wearable technologies, and more extensive biological sampling. Nevertheless, a future collaboration between these two approaches stands to accelerate the understanding of neurodegenerative disorders.

## Conclusion

This phenotype-agnostic, population-based, bio-subtyping and bioassay development program will provide longitudinally-collected clinical and biological data to characterize patients affected by neurodegenerative diseases –not to understand diseases, but to understand how individuals are affected by them. The inclusivity and large number of deeply-phenotyped individuals (currently classified under a range of neurodegenerative disorders) and the causal model-driven nature of analyses, blinded to the clinical disease classification, are unique elements in the design of this study, expected to identify small but molecularly suitable subsets of subjects for embedded proof-of-concept adaptive clinical trials. Our goal is to identity the first molecular subset of individuals for whom an available therapy can be repurposed before the end of the 2020s. Despite many anticipated challenges, the ascertainment of biological subtypes will help to materialize the promise of precision medicine for patients affected by neurodegenerative disorders.

## Author Contributions

AS organized, executed the research project, conceived and wrote the first draft of the manuscript. LM and MK organized, executed the research project and critically revised the manuscript. AKD and LL conceived the statistical methods and critically revised the manuscript. JV, APD, PL, MP, BW, EH, BS, EK, AV, LW, DBH, MR, CT, DWH, SE, KE, and RF critically revised the manuscript. ML organized the research project, conceived the statistical methods and critically revised the manuscript. AE organized, executed and supervised the research project, conceived and wrote the first draft of the manuscript. All authors contributed to the article and approved the submitted version.

## Conflict of Interest

AKD is currently supported as a co-investigator by the NIH (1 R21 HL143030-01) and (R21 AI133207) grants. He is also currently serving as a statistician in CPRIT funded studies (PP200006, PP190058, PP180003, and PP170068). The author is also an Adjunct Associate Professor in the department of neurology and rehabilitation medicine, University of Cincinnati. MK is an employee of the CONICET. He has received grant support from Ministry of Science and Technology of Argentina and Ministry of Health of Buenos Aires. Genetics and metabolism (Elsevier, Inc., New York, NY, United States), and PLOSone (Public Library of Science, San Francisco, CA, United States). BS has been paid by Aligning Science Across Parkinson’s Workshop, Chan Zuckerberg Initiative’s Neurodegeneration Challenge Network Meeting, StemCellTalks Toronto, Partnerships in Clinical Trials Europe 2018, 2019 World Parkinson’s Congress, 2019 ADPD Congress – Roche sponsored talk, Alkahest, Biolegend, Lysosomal Therapeutics, Abbvie’s Sharing for Better Caring Symposium, University of Toronto Neuroscience Rounds, McGill (multiple classes), EPFL Open Science Initiative, University of Cincinnati Neuroscience Rounds, Tanenbaum Open Science Initiative, 2018 and 2019 Rallying to the Challenge at Grand Challenges in Parkinson’s Symposium, Parkinson’s Canada Research Symposium, European Parkinson’s Disease Association’s YOPD Symposium at the European Union Headquarters, 2018 Synuclein Meeting, 2018 Ontario Brain Institute Research Summit, The Buck Institute, 23andMe, System1 Biosciences, Duke-NUS, NIH Neuroscience Rounds, Aspen Biosciences, Biogen, Zambon, Lundbeck, Cerevel, Idorsia. He is a member of the patient advisory board for the Toronto Western Hospital’s Movement Disorder Clinic and a contributing editor to the Journal of Parkinson’s. AV received funding from R01 NIH/NINDS NS103824-01, R01 NINDS NS100417, NIH/NINDS 1U01NS100699, R01 NIH/NINDS NS30678, Imaging Core Lab, ENDOLOW Trial, Cerenovus, Johnson and Johnson, Human centered design grant, and ACR Innovation Fund. CT is an employee of the University of California – San Francisco and the San Francisco Veterans Affairs Health Care System. She receives grants from the Michael J. Fox Foundation, the Parkinson’s Foundation, the Department of Defense, BioElectron, Roche/Genentech, Biogen Idec and the National Institutes of Health, compensation for serving on Data Monitoring Committees from Voyager Therapeutics, Intec Pharma and Cadent Therapeutics and personal fees for consulting from Neurocrine Biosciences, Adamas Therapeutics, Gray Matter, Acorda, Acadia, Amneal and CNS Ratings. SE is co-founder of and shareholder in evox Therapeutics that develops engineered exosome therapies to treat genetic diseases. ML received grant support from the Michael J. Fox Foundation. AE has received grant support from the NIH and the Michael J. Fox Foundation; personal compensation as a consultant/scientific advisory board member for Abbvie, Neuroderm, Neurocrine, Amneal, Adamas, Acadia, Acorda, InTrance, Sunovion, Lundbeck, and USWorldMeds; publishing royalties from Lippincott Williams and Wilkins, Cambridge University Press, and Springer; and honoraria from USWorldMeds, Acadia, and Sunovion. He serves as Associate Editor of the Journal of Clinical Movement Disorders and on the editorial boards of JAMA Neurology, the Journal of Parkinson’s Disease and Parkinsonism and Related Disorders. The remaining authors declare that the research was conducted in the absence of any commercial or financial relationships that could be construed as a potential conflict of interest. The reviewer SS declared a past co-authorship with one of the authors, AE, to the handling Editor.

## Footnotes

1 https://epi.grants.cancer.gov/DHQ/about/
